# Mismatch between Probiotic Benefits in Trials versus Food Products

**DOI:** 10.3390/nu9040400

**Published:** 2017-04-19

**Authors:** Mary J. Scourboutakos, Beatriz Franco-Arellano, Sarah A. Murphy, Sheida Norsen, Elena M. Comelli, Mary R. L’Abbé

**Affiliations:** 1 Department of Nutritional Sciences, Faculty of Medicine, University of Toronto, Toronto, ON M1E 3S1, Canada; m.scourboutakos@mail.utoronto.ca (M.J.S.); beatriz.francoarellano@mail.utoronto.ca (B.F.-A.); sarah.murphy@mail.utoronto.ca (S.A.M.); sheida.norsen@alum.utoronto.ca (S.N.); 2 Center for Child Nutrition and Health, Faculty of Medicine, University of Toronto, Toronto, ON M1E 3S1, Canada

**Keywords:** probiotics, yogurt, functional foods, microbiome, dairy products, food supply, packaged foods, Canada, public health, preventive medicine

## Abstract

Probiotic food products contain a variety of different bacterial strains and may offer different health effects. The objective was to document the prevalence and dosage of probiotic strains in the Canadian food supply and to review the literature investigating these strains in order to understand what health benefits these products may offer. The Food Label Information Program was used to identify probiotic-containing products in the food supply. PubMed, Web of Science, and Embase were searched for randomized controlled trials that tested the health effects of these strains in humans. There were six probiotic strains/strain combinations identified in the food supply. Thirty-one studies investigated these strains and found that they are associated with decreased diarrhea and constipation, improved digestive symptoms, glycemic control, antioxidant status, blood lipids, oral health, and infant breastfeeding outcomes, as well as enhanced immunity and support for *Helicobacter pylori* eradication. There were a limited number of studies investigating these strains. Many studies were funded by the food industry and tested dosages that were up to twenty-five times the dosage found in most food products. Probiotic food products could have health benefits not currently reported on their labels. However, many dosages are too low to provide the benefits demonstrated in clinical trials. Further research is needed to enable more effective use of these functional foods.

## 1. Introduction

Probiotics are “live microorganisms that when administered in adequate amounts confer a health benefit on the host” [1,2]. The benefits of consuming bacteria have been known since ancient times, when fermented milk was commonly prescribed to treat an upset stomach [3]. Today, the term “probiotic” has been defined and qualified by the World Health Organization, which put also forward guidelines to support their use. Accordingly, different probiotics have been shown to prevent or treat a wide range of health issues, including respiratory tract infections, infectious diarrhea, atopic eczema associated with cow’s milk allergy, infant colic, necrotizing enterocolitis, pouchitis, bacterial vaginosis, *Clostridioides* (formerly *Clostridium*) *difficile*-associated diarrhea, and urinary tract infections [4,5,6].

Probiotic food products are one of the fastest growing product markets globally [7]. Currently, commercial probiotic food products contain a variety of different probiotic species and strains. Certain health benefits are common to most or all probiotic species. These effects are considered “core benefits” and include the regulation of intestinal transit, normalization of perturbed microbiota, turnover of enterocytes, competitive exclusion of pathogens, colonization resistance, and short-chain fatty acid production [2]. Meanwhile, some probiotic effects are found only among specific species of probiotics. Examples include vitamin synthesis, gut-barrier reinforcement, bile salt metabolism, enzymatic activity, and neutralization of carcinogens [2]. Lastly, certain benefits may only be found among specific strains of bacteria; this includes neurological effects, immunological effects, endocrinological effects, and the production of bioactives [2].

Therefore, probiotic food products currently in the marketplace may have the potential to offer a variety of different health benefits, depending on the specific species and strains of bacteria they contain. However, depending on in which country the products are being sold, consumers have varying degrees of information about the health benefit a probiotic product has been designed to provide.

The WHO has recommended that, where scientific evidence exists, strain specific probiotic health claims should be allowed to enable the linkage of a product to a specific health effect [8]. However, in the European Union, there are no approved probiotic health claims [9]. In fact, even the word “probiotic” is considered a health claim and is not permissible on food packages. In the US, products containing probiotics can state that they ”support” the body or ”maintain” general well-being (for instance, some products state; ”help support your immune system” or ”helps naturally regulate the digestive tract”) [10]. Meanwhile, in Canada, products contain a general health claim (such as “promotes a healthy gut flora”) but could provide more specific benefits depending on the species and strain(s) they contain [8,11,12].

To date, the majority of systematic reviews investigating probiotics have focused on the effects of different strains on a single health outcome or the effects of a single strain on different health outcomes. Furthermore, there have been no reviews focused exclusively on probiotics delivered in food formats.

This study had two objectives; first, to document the prevalence and dosage of probiotic species/strains in the Canadian food supply and, second, to review the literature investigating these species/strains in order to understand what health benefits consumers could potentially receive from the probiotic products in the marketplace.

## 2. Materials and Methods

### 2.1. Investigation of Probiotic Strains in the Food Supply

Data was derived from the Food Label Information Program (FLIP), a database of Canadian food package label information derived from major outlets of the three largest grocery chains in Canada (Loblaws, Metro, and Sobeys) and one major western retailer (Safeway) [13]. This database represents 75.4% of the grocery retail market share in Canada [14] and provides a detailed assessment of the nutrition information found on Canadian packaged food labels. Grocery store shelves were systematically scanned, and data for every food product with a Nutrition Facts table (NFt), including all available national and private label brands, were collected. Data for food products sold at multiple retailers were collected only once. When multiple sizes of a product were available, only one size was collected. However, all flavors and varieties of a product were collected. Information collected for each product included the Universal Product Code, company, brand, price, Nutrition Facts table information (serving size, calories etc.), ingredients, container size, nutrient content claims, disease risk reduction claims, function claims, front of pack symbols, children’s marketing, and other claims (e.g., organic, natural, and gluten-free), in addition to the date and location of sampling. The FLIP database is updated every three years. Presently, two collections have been completed (in 2010 and 2013) and have been described in greater detail elsewhere [13,15]. The packages were visually inspected, and ingredient lists of the 15,341 unique products collected in 2013 were searched to identify probiotic-containing products. Fermented foods were not considered to be probiotic products unless they were labeled as being probiotic. The species, strain(s), and dosage found in the 92 probiotic-containing products were recorded and tabulated. In July 2016, Loblaws, Metro, and Sobeys were revisited to identify if any probiotic strain and dosage information had changed and to investigate if new probiotic products had entered the marketplace. Four new probiotic products were identified and included in this study. Companies that listed species names without strain information were contacted via e-mail to inquire whether strain data could be disclosed. One company provided strain information via e-mail.

### 2.2. Review of Randomized Controlled Trials Testing the Probiotic Strains Found in the Canadian Food Supply

A systematic search of the peer-reviewed literature investigating each strain/strain combination found in the food supply was conducted in 2016 in accordance with the preferred reporting items for systematic reviews and meta-analysis protocols (PRISMA) checklist (with the exception of items related to meta-analyses) [16]. The full detailed protocol for this is available at PROSPERO registry CRD42106042660 [17].

#### 2.2.1. Eligibility Criteria

##### Study Design, Treatment, and Participants

Double-blind randomized-controlled trials that tested the effects of probiotic strains in the food supply were considered. The probiotic strains were required to be administered in a food format similar to the formats found in the food supply.

Studies that administered probiotics in supplement form, that tested synbiotics, or investigated the safety, tolerance, persistence, or viability of probiotics were not included.

Studies on humans of all ages were considered, with the exception of infants under six-months. Individuals with a chronic disease (like diabetes), infections (such as *Helicobacter pylori*), or conditions (like constipation or Irritable Bowel Syndrome) were included.

##### Outcome Measures

This was not a traditional systematic review. This was an exploratory review and descriptive synthesis that aimed to understand what health effects these food products may offer. Therefore, any and all health-related outcome measures in humans were recorded. This ranged from serum lipid and glycemic levels, to incidence/duration of infections and illness, to markers of inflammation. Effects detected in-vitro were not included. Effects on cellular immunomodulation (e.g., increased number of lymphocytes) were not included.

#### 2.2.2. Literature Search

PubMed, Web of Science, and Embase were searched by two independent reviewers (Beatriz Franco-Arellano and Sarah Murphy) from the earliest record to July 2016. The following keywords were searched in the title/abstract: (multiple iterations of each strain name) and (yogurt OR yoghurt OR milk OR fermented milk OR dairy) with (randomized controlled trial) in any field. When the strain was not found in a dairy product, the dairy keywords were omitted. The search was limited to full-manuscripts in English. Only randomized controlled trials in humans were searched.

##### Study Selection

After the removal of duplicates, two independent reviewers (Beatriz Franco-Arellano and Sarah Murphy) screened the title and abstracts of retrieved studies against the a priori selection criteria. The selection criteria included any double-blind randomized controlled trial reported in a peer-reviewed journal that included strain/strain combinations found in the food supply, a control group, a quantified dose of the probiotic, a quantified measure of the food treatment, and oral administration of the probiotic via a food format. The study could test any clinical health endpoint on any human population (healthy or sick, including pregnant and breastfeeding mothers). Full-text screening was completed independently by two of the authors of this paper (Mary Scourboutakos and Sarah Murphy), with consensus required for inclusion or exclusion.

##### Data Extraction

The following information was extracted from each manuscript; information related to the article (complete citation plus author, country, and year of publication), the probiotic species and strain(s) tested, strain dosage, food format, population characteristics (e.g., adults, children, male, female, both), health status of the population (e.g., healthy, population with constipation, diabetic population), sample size, study duration, primary outcome measure, secondary outcome measure(s), significant outcomes, and source of funding. Data was independently extracted by one author (Mary Scourboutakos) and verified by a different author (Sarah Murphy). When articles reported insufficient information, attempts were made to contact their authors via-e-mail to retrieve further information.

##### Assessment of Methodological Quality

The study quality was independently assessed by one author (Mary Scourboutakos) using Health Canada’s quality appraisal tool for intervention studies [18] and independently checked by another author (Sarah Murphy). This tool is used to evaluate the quality of studies that provide evidence to support health claim submissions. The risk of bias was assessed using the Cochrane risk-of-bias tool (Table A1) [19].

##### Data Synthesis

All studies were grouped according to the strain/strain combination they investigated, and health outcomes were recorded accordingly.

## 3. Results

**Figure 1:**
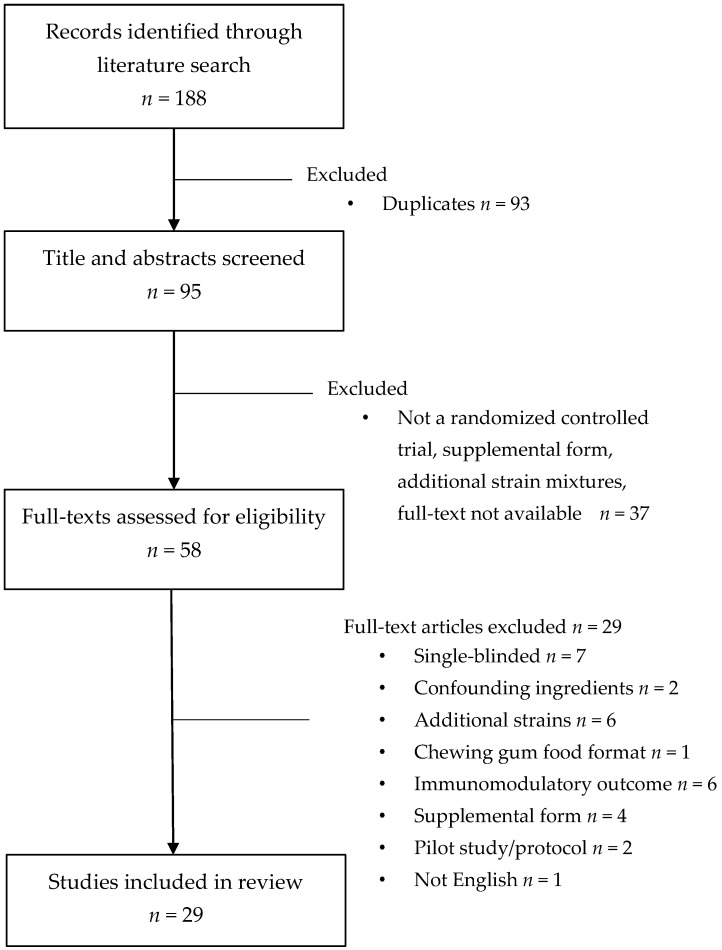
Identification of eligible studies.

The probiotic strains found in the Canadian food supply and a summary of their health effects are shown in Table 1. The initial search of the probiotic strains found in the food supply and their health benefits yielded 188 papers, with 95 remaining after the removal of duplicates (Figure 1). After reviewing the titles and abstracts, 59 remained for full-text review, 29 of which were eligible for inclusion (Table 2). All studies were deemed to be of a ‘high quality’ according to Health Canada’s quality appraisal tool for intervention studies. The majority of studies were judged to have an overall low risk of bias (Table A1).

Danone’s *DanActive* contained one proprietary strain (*Lactobacillus casei* DN 114-001). This was one of the most well studied strains in the food supply with eleven studies, all funded by Danone, investigating its effects [35]. Three studies showed decreased incidence [26,35] and duration [36] of common infectious diseases (ranging from upper respiratory tract infections to sore throats and influenza) in adults, children, and seniors. Of these, one study showed decreased duration of acute diarrhea in children [31]. One study of hospitalized elderly adults showed decreased incidence of *Clostridium difficile* and antibiotic-associated diarrhea [33]. Other effects associated with this strain included decreased asthma and rhinitis episodes [30] and increased *Helicobacter pylori* eradication rates in children [34]. One study tested the effect of this strain when consumed by breastfeeding mothers and showed that their infants had a reduced incidence of gastrointestinal episodes and a lower rate of medication use [37]. The probiotic dosage administered in these studies was up to three times the dosage found in one serving of this product.

Danone’s *Activia* contained a different proprietary strain, *Bifidobacterium lactis* DN-173 010. This strain was associated with improved overall GI well-being, including decreased flatulence [38], decreased stomach rumbling, and improved stool consistency [39]. In one study of women with irritable bowel syndrome (IBS), this strain was shown to decrease overall IBS symptom severity and to decrease maximal abdominal bloating [40].

President’s Choice’s *ProAdvantage* contained *Lactobacillus acidophilus* NCFM. One study tested this strain in children and found decreased incidence of fever, cough, rhinorrhea, antibiotic use, symptom duration, and days missed from school [42]. However, the dosage tested in the study (20 billion colony forming units (cfu) per day) was twenty times the dosage found in the product (1 billion cfu per day). Astro’s *BioBest* contained *Lactobacillus acidophilus* NCFM in combination with *Bifidobacterium lactis* Bi-07. This combination was tested in the same study reported above and was found to have the same effects and dosage discrepancy.

*Bifidobacterium lactis* BB-12 was found in two brands; Iogo’s *Probio* and Yoplait’s *Minigo* (a product intended for children). This strain was investigated in four studies. In one study, testing a dosage that was half of what is found in these products, this strain was associated with decreased levels of a cavity causing bacteria (*mutans streptococci*) in saliva [29]. Two studies tested the effect of this strain (at a dosage that was ten-times the dosage found in the product) on children’s risk of illness and absences from school [27,48]. No effects were seen. One study tested a dosage that was thirty-five times the dosage found in the products containing this strain and showed no effect on inflammatory markers (C-reactive protein and cytokines) [28].

*Bifidobacterium lactis* BB-12 in combination with *Lactobacillus acidophilus* LA-5 was found in two brands (Yoplait’s *Yoptimal* and Lucerne’s *Organics*). Eleven studies investigated this strain combination. Three studies tested dosages that were substantially smaller than the dosage found in commercial products. Two of those studies showed reduced salivary levels of cavity causing bacteria (*Streptococcus mutans*) [24,25], while one showed decreased duration of antibiotic-associated diarrhea in patients infected with *Helicobacter pylori* [20]. Two studies investigated the impact of these strains on blood lipids and found no effects despite the fact that one study tested a dosage that was lower than found in commercial products and the other tested a dosage that was higher [22,23]. One study investigated the effect of these strains on glycemic control [21]. It tested a dosage that was three-times the dosage found in commercial products and found decreased insulin sensitivity. There were four studies that tested the effect of these strains on type-two diabetics and used dosages that were similar to those found in commercial products (Table 3). These studies showed improved glycemic control [43,47], improved blood lipid levels [44,45,47], and enhanced antioxidant status [43] in diabetics.

Nine brands labeled species names without identifying the strain (Table 4). Therefore, strain-specific health benefits could not be inferred for these products.

Most products contained one or two different strains. Kefir (here a fermented milk with added probiotics) products had the largest strain and species diversity, as well as the highest dosage (45 billion colony forming units per serving). However, not all kefir products contained this dosage and diversity.

## 4. Discussion

Probiotic food products in the Canadian marketplace contained bacterial strains that were associated with a wide variety of health benefits ranging from enhanced immunity to improved glycemic control in diabetics, suggesting that probiotic products could potentially offer health benefits that are not advertised on their labels. However, many of the current probiotic dosages in products were lower than the dosages tested in randomized controlled trials.

### 4.1. Dosage

In order to obtain many of the health benefits reported in the randomized controlled trials that were reviewed in this study, consumers would need to eat anywhere from two to twenty-five servings of these products each day. The WHO has recommended that “the suggested serving size (on the product label) must deliver the effective dose of probiotics related to the health claim” [8]. Currently most products contain one billion colony forming units (CFU) of probiotics because that is the minimum required in order to provide core benefits and thus be eligible to display the probiotic health claim “promotes a healthy gut flora” in Canada [12]. Therefore, if strain-specific health claims were implemented (in addition to the existing general probiotic health claim), companies would have greater incentive to provide the higher dosages needed to convey some of the health benefits reported in this review.

### 4.2. Strain Diversity

Most products contained one or two strains. However, research has shown that, in some cases, strain mixtures can be more effective than single strains [50,51,52,53,54], as “different strains (that are) targeted toward different ailments can be blended into one preparation”, enabling cultures to complement each other’s health effects and produce synergistic benefits [55]. For instance, *Bifidobacterium lactis* BB-12 (found in the food supply) has been shown to have greater gut-adherence when accompanied by *Lactobacillus rhamnosus* GG (one of the most well-studied probiotic strains [4], which is mainly available in supplement form) [56]. Furthermore, evidence from Leyer et al.’s investigation of *Lactobacillus acidophilus* NCFM alone and in combination with *Bifidobacterium lactis* Bi-07 showed that the combination of strains resulted in a lower risk of fever, coughing, and rhinorrhea when compared to the single strain [42]. It is understandable that our results found that fewer products contained strain mixtures, as, presently, food companies have no incentive to utilize strain synergies. Not to mention that single strain probiotics are more easily patentable than multi-strain probiotics [52]. Therefore, current health claims that are based on a single strain encourage the addition of single strains and could therefore be partially responsible for promoting a potentially suboptimal pharmaceutical-like approach to probiotic foods. That being said, it should be noted that not all strain mixtures are beneficial, as strains can antagonize one another. Therefore, research is needed to verify if mixtures are synergistic or antagonistic [50]. It has been previously noted that there is a lack of research on multi-strain probiotics because such research is more difficult to conduct and thus more expensive [57].

### 4.3. Strengths and Limitations

A strength of this study is the use of FLIP to derive information on all marketed products, which is why we chose to focus on the Canadian market as a model. Obviously, different markets will have different probiotic-containing products, which may come with benefits that overlap or differ from those discussed here. It is important that future research focuses on these other markets to the benefit of both the consumers and the industry. Furthermore, many probiotic benefits could vary depending on an individual’s lifestyle and baseline microbiome. Therefore, it is expected that the health effects noted in this review may not benefit all consumers equivalently.

Limitations include the fact that studies in this review tested various strains, dosages, and health outcomes. Therefore, at this point in time, there is no consensus on what strain, dose, or product is best. For example, while our review showed that one strain (*Bifidobacterium lactis* DN-173 010) was associated with decreased digestive symptoms, this was the only strain for which this outcome was assessed. Hence, we cannot conclude that other strains/products would not also have these benefits. Therefore, these results show what is known according to the limited amount of literature that currently exists. Additionally, since much of the current research was funded by the companies that sell probiotic products and therefore dictated which strains were studied, there is a need for further research on a broader range of species/strains that is supported by alternate funding bodies.

Despite the WHO’s recommendation that genus, species, and strain should be designated on a product’s label [8], nearly half of the brands in this study did not disclose strain information. Thus, the potential health benefits for these products could not be deduced.

Previous research has shown that industry funded nutrition-related research may bias conclusions in favor of the sponsors’ products [58]. Most of the studies included in this review were funded by the companies making the products [26,30,32,33,34,35,36,37,40,59] or were published in journals that are funded by the food industry (Table 1) [45,46]. Many of these studies investigated a large number of outcome measures but did not make statistical adjustments to control for testing multiple hypotheses. Furthermore, in many of these studies, the primary outcome measure was not significant, and, instead, significance was detected in secondary outcomes or through post-hoc analyses. Therefore, while these studies were deemed to be of high quality and were published in peer-reviewed journals, elements of their analysis suggest that their results should be interpreted with caution.

## 5. Conclusions

Probiotic food products sold in Canada could offer a variety of health benefits depending on the strain(s) and dosage they contain. That being said, the probiotic dosages contained in most food products are currently too low to provide the benefits shown in clinical trials. Therefore, with higher dosages, or with trails substantiating the current dosage, there is potential for the strains that are already in food products to provide more benefits to the consumer.

Currently there is only a small volume of literature investigating the health benefits of the probiotic strains used in the Canadian food supply. Thus, additional clinical trials, particularly ones that are not sponsored by the food industry, are needed. Hopefully this work will encourage funding and regulatory agencies to fund more research investigating probiotics. A larger number of well conducted studies and clear evidence-based labeling regulations will ultimately help the consumers to make informed choices and derive substantiated benefits form the products they choose to consume.

Overall, considering the wide range of diseases and health conditions for which probiotics have been shown to have benefits, further research to promote the optimal design of probiotic food products is needed to enable more effective use of these functional foods.

## Figures and Tables

**Table 1:** Strains in probiotic food products and reported health effects associated with these strains.

Strain(s)	Manufacturer and Product Brand	Food Type	Probiotic Dosage in Food (CFU */Serving)	Dosage Tested in Studies (CFU */Day)	Duration of Study	Health Effects Investigated in Healthy Populations										
						Acute Diarrhea	Antibiotic-Associated Diarrhea	Constipation	Digestive Symptoms	Glycemic Control	*Helicobacter pylori* Eradication	Immunity	Infant Breastfeeding Outcomes	Inflammation	Serum Lipids/Blood Pressure	Oral Health
*Bifidobacterium lactis* BB12 + *Lactobacillus acidophilus* LA-5	Yoplait’s *Yoptimal*, Lucerne’s *Organics* †	Yogurt	>1 × 10^9^	2 × 10^6^–3 × 10^9^	7 days–6 weeks		X [20] ^$^			O [21]	O [20] ^$^				O [22,23]	x [24,25]
*Bifidobacterium lactis* BB12	Iogo’s *Probio* **, Yoplait‘s *Minigo*	Yogurt	>1 × 10^9^	1 × 10^10^–3.5 × 10^10^	10 days—3 months							o [26] ^$^ [27]		O [28] ^$^		X [29]
*Lactobacillus casei* DN 114-001	Danone’s *DanActive*	Drinkable yogurt	1 × 10^10^	1 × 10^10^–3 × 10^10^	2 weeks –6 months	x [30] ^$^, [31] o [32] ^$^	X [33] ^$^				x [34] ^$^	X [35,36] ^$^ x [26,30] ^$^	x [37] ^$^			
*Bifidobacterium lactis* DN-173 010	Danone’s *Activia*	Yogurt	>1 × 10^9^	8 × 10^9^–2.5 × 10^10^	2–4 weeks			o [38] ^$^	X [39,40] ^$^ x [38] ^$^							O [41] ^$^
*Lactobacillus acidophilus* NCFM + *Bifidobacterium lactis* Bi-07	Astro’s *BioBest*	Yogurt	1 × 10^9^	2 × 10^10^	6 months	o [42] ^$^						x [42] ^$^				
*Lactobacillus acidophilus* NCFM	President’s Choice’s *ProAdvantage* †	Yogurt	1 × 10^9^	2 × 10^10^	6 months	o [42] ^$^						x [42] ^$^				

X = beneficial effects observed in healthy adults; x = beneficial effects observed in healthy children, O = studies that have investigated this outcome and have found no significant effect in adults, o = studies that have investigated this outcome and found no significant effect in children, ^$^ = indicates that the research was funded by the company that uses that particular strain in their products. A blank square indicates that no research investigating the effects of that strain/strain combination was identified during the systematic review of all literature published up to 21 July 2016, as described in the methods. All effects reported in this table were found in healthy populations that were not diagnosed with a chronic disease or condition. Definition of health effects: Constipation = improved stool frequency, consistency, or condition; Acute diarrhea = decreased incidence or severity of acute diarrhea; Antibiotic-associated diarrhea = decreased incidence of antibiotic-associated or *Clostridium difficile*-associated diarrhea; Digestive symptoms = decreased abdominal pain/discomfort, bloating, flatulence, or overall GI well-being; Glycemic control = improved fasting glucose, insulin, HbA1c (marker of long-term glycemic control), or HOMA-IR (measure of insulin sensitivity); *Helicobacter pylori* eradication = enhanced eradication of *Helicobacter pylori* infections; Immunity = decreased incidence and/or duration of common infectious diseases, including fever, cough, common respiratory infections (rhinitis, sore throat), common gastrointestinal infections (gastroenteritis, vomiting), asthma, or days missed from school; Infant breastfeeding outcomes = infants (2–6 months old) of mothers who consume this strain while breastfeeding had decreased incidence of gastrointestinal episodes and lower medication-use rates; Inflammation = decreased levels of inflammatory markers (ex. C-reactive protein); Lipids = decreased serum total cholesterol, low density lipoprotein (LDL), triglyceride levels, or increased high density lipoprotein (HDL); Oral health = decreased levels of cavity causing bacteria. * CFU = colony forming units. ** Iogo’s *Probio* reported two strains on its label in 2013 (*Bifidobacterium lactis* BB12 + *Lactobacillus acidophilus* LA-5) and only one strain on its label in 2016. † These products were available in 2013 but may no longer be available in the Canadian market. Note: All cited references were deemed to be of high quality according to Health Canada’s quality appraisal tool for intervention studies [18].

**Table 2:** Results of the review of randomized controlled trials investigating the health effects of probiotic strains found in the Canadian food supply ^1^.

Strain	Study, Country (Year)	Population (*n*)	Probiotic Dosage (CFU per Day)	Study Duration	Outcome Measures (Primary and Secondary)	Statistically Significant Effects (Relative to Placebo Group)	Funding Source
*B. lactis* BB12 + *L. acidophilus* LA-5	Ivey et al. [21] Australia (2014)	Overweight adults *n* = 156	3 × 10^9^	6 weeks	**Primary:** Glycemic control (fasting blood glucose, insulin, HbA1c, and HOMA-IR)	Increased HOMA-IR (worsened insulin sensitivity)	Sir Charles Gairdner Hospital
	Sadrzadeh-Yeganeh et al. [23] Iran (2010)	Females *n* = 90	3.9 × 10^7^	6 weeks	**Primary:** Serum total cholesterol, HDL, LDL, and triglycerides	No observed effects	Tehran University Grant
	Ivey et al. [22] Australia (2015)	Overweight adults *n* = 156	3 × 10^9^	6 weeks	**Primary:** Blood pressure, total cholesterol, HDL, LDL, and triglycerides	No observed effects	Sir Charles Gairdner Hospital
	deVrese et al. [20] Germany (2011)	H pylori infected adults *n* = 88	5 × 10^6^	5 weeks	**Primary:** *Helicobacter pylori* activity; **Secondary:** Frequency, intensity and duration of abdominal pain; stool frequency/consistency; duration of diarrhea episodes; IBS symptoms; orofecal transit time	Decreased duration of antibiotic-associated diarrhea episodes	Chr. Hansen GmbH J. & Co., KG, NOM AG ^$^
	Ashwin et al. [24] India (2015)	Children *n* = 60	2 × 10^6^	7 days	**Primary:** Salivary levels of streptococcus mutans (a cavity causing bacteria)	Reduced salivary *mutans streptococci*	Funded by study author
	Singh et al. [25] India (2011)	Children *n* = 40	5.4 × 10^7^	10 days	**Primary:** Salivary levels of salivary *mutans streptococci* and *lactobacilli* (cavity causing bacteria)	Reduced salivary *mutans streptococci*	Not disclosed
	Ejtahed et al. [43] Iran (2011)	Type II Diabetics *n* = 64	>1 × 10^9^	6 weeks	**Primary:** Fasting blood glucose, HbA1c, insulin and antioxidant molecules (superoxide dismutase, glutathion peroxidase, catalase activity, malondialdehyde concentration, and total antioxidative status)	Decreased fasting blood glucose and HbA1c; increased activity of superoxide dismutase, glutathoine peroxidase, and total antioxidative status	Iran Dairy Industry ^$^
	Mohamadshahi et al. [44] Iran (2014)	Type II Diabetics *n* = 44	>1 × 10^9^	8 weeks	**Primary:** Serum triglycerides, LDL, HDL, triglycerides, LDL:HDL	Decreased LDL:HDL, increased HDL	Nutrition Disease Research Center
	Ejtahed et al. [45] Iran (2012)	Type II Diabetics *n* = 60	6 × 10^8^	6 weeks	**Primary:** total cholesterol, triglycerides, HDL, LDL, total cholesterol:HDL, LDL:HDL	Decreased total cholesterol, LDL, LDL:HDL and total cholesterol:HDL	Grant from Tabriz University
	Nabavi et al. [46] Iran (2014)	Non-alcoholic fatty liver disease patients *n* = 72	>1 × 10^9^	8 weeks	**Primary:** Blood levels of liver enzymes (alanine aminotransferase and aspartate aminotransferase); fasting blood glucose; total cholesterol, triglycerides, LDL, HDL.	Decreased blood levels of liver enzymes, total cholesterol, triglycerides, and LDL	Nutrition Research Center, Tabriz University
	Tonucci et al. [47] Brazil (2015)	Type II Diabetics *n* = 45	2 × 10^9^	6 weeks	**Primary:** Glycemic control (fasting blood glucose, insulin, HOMA-IR, fructosamine, HbA1c); lipid profile (total cholesterol, LDL, VLDL, triglycerides, total cholesterol:HDL); total antioxidant status and cytokine concentrations (Il-6, Il-10, TNF-α, adiponectin, and resistin); fecal short-chain fatty acids	Decreased fructosamine, LDL, and total cholesterol; significant change in HbA1c	Brazilian Agri-Research; Foundation to Support the State of Miras Gerais
*B. lactis* BB12	Caglar et al. [29] Turkey (2008)	Healthy young adults *n* = 24	5 × 10^8^	10 days	**Primary:** Salivary levels of *mutans streptococci* and *lactobacilli* (cavity causing bacteria)	Decreased salivary *mutans streptococi*	Funded by researchers
	Merenstein et al. [48] USA (2010)	Children *n* = 182	1 × 10^10^	90 days	**Primary:** Missed days of school due to illness; **Secondary:** Diarrhea, stool consistency, respiratory infection, missed parental work, doctor visits, illnesses, and overall parental satisfaction	No observed effects	The Gerber Foundation ^$^
	Merenstein et al. [27] USA (2011)	Healthy children *n* = 172	1 × 10^10^	90 days	**Primary:** Missed days of school due to illness; **Secondary:** Diarrhea, stool consistency, respiratory infection, missed parental work, doctor visits, illnesses	No observed effects	USDA
	Kekkonen et al. [28] Finland (2008)	Healthy adults *n* = 62	3.5 × 10^10^	3 weeks	**Primary:** Blood levels of inflammatory markers including C-reactive protein and cytokines (TNF-α, IL-6, IFN-γ, IL-10)	No observed effects	Resaerch Council Finland and Valio ^$^
*L. acidophilus* NCFM + *B. lactis* Bi-07	Leyer et al. [42] China (2009)	Healthy children *n* = 326	2 × 10^10^	6 months	**Primary:** Frequency and duration of fever, cough, rhinorrhea, vomiting, diarrhea, physicians’ visits and antibiotic prescriptions; **Secondary:** School absences	Decreased incidence of fever, cough, rhinorrhea, antibiotic use, and days missed from school. Reduced symptom duration.	Danisco ^$^
*B. lactis* DN-173 010	Pinto et al. [41] Brazil (2013)	Healthy adults *n* = 26	not reported	2 weeks	**Primary:** Salivary levels of cavity-associated microorganisms (*mutans streptococci*, *lactobacilli* and total microorganisms) in saliva	No observed effects	Not Disclosed
	Tabbers et al. [38] Netherlands and Poland (2011)	Constipated children *n* = 159	>8 × 10^9^	3 weeks	**Primary:** Stool frequency; **Secondary:** Stool consistency, frequency of faecal incontinence, pain during defecation, abdominal pain, flatulence	Decreased flatulence	Danone ^$^
	Guyonnet et al. [39] Germany (2009)	Healthy adult women *n* = 192	2.5 × 10^10^	4 weeks	**Primary:** Overall GI well-being (intestinal transit, stool frequency and consistency, abdominal pain/discomfort, bloating, flatulence, stomach rumbling); **Secondary:** Frequency of digestive symptoms including abdominal pain/discomfort, bloating, flatulence, stomach rumbling; stool frequency and consistency; health-related quality of life	Improved overall GI well-being; decreased frequency of flatulence, stomach rumbling, improved stool consistency, and health-related quality of life.	Danone ^$^
	Agrawal et al. [40] United Kingdom (2008)	Adult females with IBS *n* = 34	2.5 × 10^10^	4 weeks	**Primary:** Abdominal distension and bloating; **Secondary:** Orocaecal and colonic transit times; incidence and severity of IBS symptoms (abdominal pain/discomfort, bloating, flatulence); overall IBS symptom severity; time and consistency of bowel movements; feelings of incomplete evacuation at time of stool passage	Decreased maximal abdominal distension, orocaecal and colonic transit times, overall IBS symptom severity, and abdominal pain/discomfort.	Danone ^$^
*L. casei* DN 114-001	Guillemard et al. [35] Germany (2010)	Healthy adult shift workers *n* = 1000	>2 × 10^10^	3 months	**Primary:** Cumulative number of common infectious diseases (CID) (e.g., sore throat, sinusitus, nasal discharge, ear ache, influenza, pneumonia, cough, GI infection, diarrhea, nausea vomiting) **Secondary:** Occurrence of having at least one CID: time to first CID, severity, duration, cumulated duration; occurrence and duration of fever, sick days, medication use	Decreased occurrence and time to first CID; decreased duration of fever; decreased cumulative number of CIDs (post-hoc analysis)	Danone ^$^
	Merenstein et al. [26] USA (2010)	Healthy children *n* = 638	>2 × 10^10^	3 months	**Primary:** Change in behaviour due to illness (e.g., missed school, missed sports activity); incidence of common infectious diseases (CIDs) **Secondary:** Absences from daycare or school, missed parental work, days with diarrhea, vomiting, stomach pain, constipation, runny nose, cough, decreasing appetite, fever, rash, medication use	Decreased incidence of CID	Danone ^$^
	Guillemard et al. [36] France (2009)	Elderly adults *n* = 1072	>2 × 10^10^	3 months	**Primary:** Cumulative number of all common infectious diseases (CID) **Secondary:** The occurrence of CID (defined as the number of subjects experiencing at least one CID), duration of CID (cumulative and per episode), time to first CID, severity of CID, fever associated with CID, occurrence or duration of medication use	Decreased duration of CID episodes and cumulative duration of CID	Danone ^$^
	Sykora et al. [34] Czech Republic (2005)	Children w/H Pylori *n* = 86	1 × 10^10^	14 days	**Primary:** Eradication rate of *Helicobacter pylori* infection	Increased *Helicobacter pylori* eradication rates	Ministry of Health and Danone ^$^
	Ortiz-Andrellucchi et al. [37] Spain (2008)	Breastfeeding infants *n* = 104	3 × 10^10^	6 weeks	**Primary:** Immunomodulatory molecules in breast milk (not included in this review) **Secondary:** Infant growth and weight; incidence of gastrointestinal episodes, respiratory symptoms, medication use, allergies and dermatitis	Reduced incidence of gastrointestinal episodes and lower rate of medication use in infants	Danone ^$^
	Agarwal et al. [31] India (2002)	Children *n* = 150	2–3 × 10^10^	9 months	**Primary:** Duration of acute diarrhea	Decreased duration of acute diarrhea	Not Disclosed
	Hickson et al. [33] United Kingdom (2007)	Elderly in-patients *n* = 137	2 × 10^10^	2 weeks	**Primary:** Incidence of antibiotic-associated diarrhea and *Clostridium difficile* associated diarrhea	Decreased incidence of antibiotic- and *Clostridium*-associated diarrhea	Danone ^$^
	Giovannini et al. [30] Italy (2007)	Children with asthma/rhinitis *n* = 187	1 × 10^10^	12 months	**Primary:** Episodes and duration of asthma and rhinitis (runny/stuff nose) **Secondary:** Episodes and duration of abdominal symtoms, diarrhea and fever	Decreased asthma and rhinitis episodes, decreased duration of diarrhea in children with rhinitis	Danone ^$^
	Giralt et al. [49] Spain (2008)	Gynecological cancer patients *n* = 85	2.8 × 10^10^	6 months	**Primary:** Frequency and severity of radiation induced diarrhea **Secondary:** Time to the development of diarrhea, stool consistency	Improved stool consistency	Danone ^$^

^1^ All probiotic strains in the Canadian food supply were recorded and a systematic review of their health effects was conducted. All literature published up to 21 July 2016 was included, as described in the methods. All studies included in the review were deemed to be of a ‘high quality’ according to Health Canada’s quality appraisal tool for intervention studies and thus are considered eligible to substantiate a health claim [18]. ^$^ Indicates that funding was provided by the food industry HbA1c = hemoglobin A1c, a long-term measure of glycemic control; HOMA-IR = a measure of insulin sensitivity; LDL = low-density lipoprotein; HDL = high-density lipoprotein; VLDL = very low-density lipoprotein; IBS = irritable bowel syndrome; CID = common infectious diseases.

**Table 3:** Strains in probiotic food products and reported health effects in populations with a diagnosed non-communicable disease/condition.

Population	Strain/Strain Combination	Manufacturer and Product Brand	Probiotic Dosage in Food (CFU */Serving)	Dosage Tested in Studies (CFU */Day)	Health Effects Investigated in Populations with a Disease/Condition						
					Antioxidant Status	Digestive Symptoms	Glycemic Control	Inflammation	Liver Damage	Radiation Induced Diarrhea	Serum Lipids
Type II Diabetics	*Bifidobacterium lactis* BB12 + *Lactobacillus acidophilus* LA-5	Yoplait’s *Yoptimal*, Lucerne’s *Organics* †	>1 × 10^9^	6 × 10^8^–>1 × 10^9^	X [43] ^$^ O [47]		X [43] ^$^, [47]	O [47]			X [44,45,47]
Patients with Non-Alcoholic Fatty Liver Disease				>1 × 10^9^					X [46]		X [46]
Females with Irritable Bowel Syndrome	*Bifidobacterium lactis* DN-173 010	Danone’s *Activia*	>1 × 10^9^	2.5 × 10^9^		X [40] ^$^					
Gynecological Cancer patients undergoing radiation therapy	*Lactobacillus casei* DN 114-001	Danone’s *DanActive*	1 × 10^10^	2.8 × 10^10^						X [49] ^$^	

X = beneficial effects observed; O = studies have investigated this outcome and have found no significant effects; ^$^ = indicates that the research was funded by the dairy industry. A blank square indicates that no research investigating the effects of that strain/strain combination was identified during the systematic review of all literature published up to 21 July 2016, as described in the methods. Effects reported in this table were observed in populations that were diagnosed with a disease or condition Definition of health effects: Antioxidant status = activity of superoxide dismutase, glutathoine peroxidase, and total antioxidant status; Digestive symptoms: decreased abdominal distension/pain/discomfort, decreased fecal transit time, reduced IBS symptom severity; Glycemic control = decreased fasting blood glucose, insulin, and/or HbA1c (long-term measure of blood glucose control); Inflammation = Increased levels of anti-inflammatory markers (cytokines: IL-6, IL-10, TNF-α, adiponectin, and resistin) Liver damage = decreased serum levels of liver enzymes (alanine aminotransferase and aspartate aminotransferase) [a marker of decreased liver damage]; Radiation Induced Diarrhea = incidence and severity; Serum Lipids = decreased serum total cholesterol, LDL, or triglyceride levels; increased HDL; improved lipid ratios. * CFU = colony forming units. † This product may no longer be available in the marketplace Note: All cited references were deemed to be of high quality according to Health Canada’s quality appraisal tool for intervention studies [18].

**Table 4:** Additional probiotic products with undetermined health effects.

**A: Products whose specific health effects are undetermined because there is no research on the strain/strain combination in their specific food format**			
Bacterial strains identified in the food database *	Manufacturer and product brand	Food type	Probiotic dosage (CFU) per serving
*Lactobacillus acidohpilus* Bi-07	Irresistibles’ *Life Smart*	Frozen fruit and yogurt blend	Not indicated
*Lactobacillus acidophilus* LA-5	Breuggens *Yog Active Cereal*	Cereal with yogurt flakes	1 × 10^9^
*Bacillus coagulans* GBI-30 6086	ShaSha Co’s *Spelt Ginger Snaps*	Cookies	Not indicated
*Lactobacillus acidophilus* ATCC 4356T	† Liberte’s *Kefir* (effervescent)	Fermented milk	4.5 × 10^10^
*Lactobacillus helveticus* ATCC 10797			
*Lactobacillus helveticus* ATCC 12046			
*Lactobacillus helveticus* ATCC 15009T			
*Lactobacillus kefir* ATCC 35411T			
*Lactobacillus kefir* ATCC 8007			
*Lactobacillus brevis* ATCC 14869T			
*Lactobacillus brevis* ATCC 13648			
*Lactobacillus kefirgranum* LMG 15132T			
*Lactobacillus parakefir* LMG 15133T			
*Lactobacillus kefiranofaciens* ATCC 43761T			
*Leuconostoc mesenteroides* ATCC 8293T			
*Leuconostoc mesenteroides* LMG 14531			
*Leuconostoc mesenteroides* LMG 6909T			
*Leuconostoc pseudomesenteroides* ATCC 12291T			
*Lactococcus lactis* subsp. *lactis* LMG 6890T			
*Lactococcus lactis* LMG 7931			
*Lactococcus lactis* subsp. *cremoris* LMG 6897			
**B: Products whose specific health effects are undetermined because they only indicate the species and not the specific strain of the bacteria they contain**			
*Bifidobacterium*	Lucerne’s *Eating Right*	Yogurt	Not indicated
*Lactobacillus casei*	Liberte’s *BioOrganic*	Yogurt	>1 × 10^9^
*Bifidobacterium lactis* + *Lactobacillus acidophilus* + *Lactobacillus casei*	Liberte’s *Classic*	Yogurt	>1 × 10^9^
	Riviera’s *Petit Pot Yogurt* **		1 × 10^9^
*Bifidobacterium lactis* + *Lactobacillus acidophilus*	Liberte’s *Goat Yogurt*	Yogurt	>1 × 10^9^
	Skotidakis’ *Greek Yogurt* **	Yogurt	not indicated
*Lactobacillus casei*	Liberte’s *Kefir* (non-effervescent)	Fermented milk	>1 × 10^9^
*Lactobacillus acidophilus*			
*Bifidobacterium lactis*			
*Lactobacillus rhamnosus*			
*Lactococcus lactis* subsp. *cremoris*			
*Lactococcus lactis* subsp. *lactis*			
*biovar diacetylactis*			
*Lactobacillus delbrueckii* subsp. *lactis*			
*Lactobacillus delbruecki* subsp. *bulgaricus*			
*lauconostoc mesenteroides* subsp. *cremoris*			
*Bifidobacterium infantis*	Iogo’s *Probio Kefir*	Fermented milk	2 × 10^9^
*Bifidobacterium lactis*			
*Lactobacillus acidophillus*			
*Lactobacillus fermentum*			
*Lactobacillus lactis*			
*Lactobacillus paracasei*			
*Lactobacillus rhamonus*			
*Lactococcus lactis* subsp. *Cremoris*			
*Lactococcus lactis* subsp. *Lactis*			
*Lactococcus lactis* subsp. *lactis biovar diacetylactis*			
*Lactobacillus delbrueckii* subsp. *bulgaricus Leuconostoc mesenteroides*			
*Leuconostoc pseudomensenteroides*			
*Bifidobacterium bifidum* + *Bifidobacterium longum* subsp *longum* + *Bifidobacterium animals* subsp. *lactis*	President’s Choice’s *Kefir* **	Fermented milk	2 × 10^9^
*Lactobacillus acidophilus* + *Bifidobacterium lactis*	President’s Choice’s *Greek Probiotic* **	Yogurt	1 × 10^9^

* Probiotic containing foods were identified in the Food Label Information Program (FLIP), a database of Canadian food package label information. FLIP data was collected in 2013 from major outlets of the four largest grocery retail chains in Canada. Probiotic species/strain information was obtained from the ingredients list and package of each probiotic product. Data was re-verified in 2016 to ensure that the species and dosage information had not changed. ** These products were not included in the 2013 database but were identified when grocery chains were revisited in 2016. † The strains associated with Liberte’s effervescent Kefir are not listed on the product label. This data was obtained via an inquiry with the company. All companies that listed species names without strains were contacted to inquire whether strain data could be disclosed.

## Appendix A

**Table A1:** Appraisal of the risk of bias of the included studies using the Cochrane risk-of-bias tool.

Strain Studied	Study	Sequence Generation	Allocation Concealment	Blinding	Incomplete Outcome Data	Selective Reporting	Overall
*Bifidobacterium lactis* BB12	Caglar et al. [29]	L	L	L	L	L	L
	Merenstein et al. [48]	L	L	L	U	L	L
	Merentstein et al. [27]	L	L	L	U	L	L
	Kekkonen et al. [28]	U	U	L	L	L	U
*Bifidobacterium lactis* DN-173 010	Pinto et al. [41]	L	L	L	L	L	L
	Tabbers et al. [38]	L	L	L	L	L	L
	Guyonnet et al. [39]	U	U	L	L	L	U
	Agrawal et al. [40]	U	U	L	L	L	U
*Lactobacillus acidophilus* NCFM + *Bifidobacterium lactis* Bi-07	Leyer et al. [42]	L	U	L	L	L	L
*Bifidobacterium lactis* BB12 + *Lactobacillus acidophilus* LA-5	Ivey et al. [21]	L	L	L	L	L	L
	Ivey et al. [22]	L	L	L	L	L	L
	Sadrzadeh-Yeganeh et al. [23]	U	U	L	U	L	U
	de Vrese et al. [20]	U	U	L	L	L	U
	Ashwin et al. [24]	U	U	L	U	L	U
	Singh et al. [25]	L	L	L	U	L	L
	Ejtahed et al. [45]	L	L	L	L	H	L
	Mohamadshahi et al. [44]	L	L	L	U	L	L
	Ejtahed et al. [43]	L	L	L	L	H	L
	Nabavi et al. [46]	L	L	L	L	U	L
	Tonucci et al. [47]	L	U	L	L	L	L
*Lactobacillus casei* DN 114-001	Guillemard et al. [36]	L	U	L	L	L	L
	Merenstein et al. [26]	L	L	L	L	U	L
	Guillemard et al. [35]	L	U	L	L	L	L
	Sykora et al. [34]	L	H	L	L	L	L
	Ortiz-Andrellucchi et al. [37]	U	L	L	L	U	U
	Agarwal et al. [31]	L	U	L	L	L	L
	Hickson et al. [33]	L	L	L	U	L	L
	Giovannini et al. [30]	L	H	L	L	L	L
	Giralt et al. [49]	L	L	L	L	L	L

Note: H = high risk of bias, L = low risk of bias, and U = unclear risk of bias.
