# Correction: Scourboutakos, M.J.; et al. Mismatch between Probiotic Benefits in Trials versus Food Products. *Nutrients* 2017, *9*, 400

**DOI:** 10.3390/nu9070641

**Published:** 2017-06-22

**Authors:** Mary J. Scourboutakos, Beatriz Franco-Arellano, Sarah A. Murphy, Sheida Norsen, Elena M. Comelli, Mary R. L’Abbé

**Affiliations:** 1 Department of Nutritional Sciences, Faculty of Medicine, University of Toronto, Toronto, ON M1E 3S1, Canada; m.scourboutakos@mail.utoronto.ca (M.J.S.); beatriz.francoarellano@mail.utoronto.ca (B.F.-A.); sarah.murphy@mail.utoronto.ca (S.A.M.); sheida.norsen@alum.utoronto.ca (S.N.); 2 Center for Child Nutrition and Health, Faculty of Medicine, University of Toronto, Toronto, ON M1E 3S1, Canada

We would like to submit the following correction to our recently published paper [1] because the wrong dose of probiotic was reported. The probiotic dosage has been corrected throughout Table 1 and Table 2 (Table 1: in the fifth column of the sixth row and in the fifth column of the seventh row, 2 × 10^10^ was changed to 1 × 10^10^; Table 2: in the fourth column of the seventeenth row, 2 × 10^10^ was changed to 1 × 10^10^). The correct tables are shown below.

These corrections induced a few minor changes in the text of the results section. As a consequence of this correction, the following sentences should be corrected:

## Results

On page 9, the second sentence of paragraph three, of the original publication [1] incorrectly stated “However, the dosage tested in the study (20 billion colony forming units (cfu) per day) was twenty times the dosage found in the product (1 billion cfu per day).”. Instead, this statement should read “However, the dosage tested in the study (10 billion colony forming units (cfu) per day) was ten times the dosage found in the product (1 billion cfu per day).”.

These changes have no material impact on the conclusions of our paper. The manuscript will be updated and the original will remain online on the article webpage. We apologize for any inconvenience caused to our readers. 

## Figures and Tables

**Table 1:** Strains in probiotic food products and reported health effects associated with these strains.

Strain(s)	Manufacturer and Product Brand	Food Type	Probiotic Dosage in Food (CFU */Serving)	Dosage Tested in Studies (CFU */Day)	Duration of Study	Health Effects Investigated in Healthy Populations										
						Acute Diarrhea	Antibiotic-Associated Diarrhea	Constipation	Digestive Symptoms	Glycemic Control	*Helicobacter pylori* Eradication	Immunity	Infant Breastfeeding Outcomes	Inflammation	Serum Lipids/Blood Pressure	Oral Health
*Bifidobacterium lactis* BB12 + *Lactobacillus acidophilus* LA-5	Yoplait’s *Yoptimal*, Lucerne’s *Organics* †	Yogurt	>1 × 10^9^	2 × 10^6^–3 × 10^9^	7 days–6 weeks		X [20] ^$^			O [21]	O [20] ^$^				**O** [22,23]	**x** [24,25]
*Bifidobacterium lactis* BB12	Iogo’s Probio **, Yoplait’s *Minigo*	Yogurt	>1 × 10^9^	1 × 10^10^–3.5 × 10^10^	10 days—3 months							o [26] ^$^ [27]		O [28] ^$^		**X** [29]
*Lactobacillus casei* DN 114-001	Danone’s *DanActive*	Drinkable yogurt	1 × 10^10^	1 × 10^10^–3 × 10^10^	2 weeks –6 months	x [30] ^$^, [31] o [32] ^$^	X [33] ^$^				x [34] ^$^	X [35,36] ^$^ x [26,30] ^$^	x [37] ^$^			
*Bifidobacterium lactis* DN-173 010	Danone’s *Activia*	Yogurt	>1 × 10^9^	8 × 10^9^–2.5 × 10^10^	2–4 weeks			o [38] ^$^	X [39,40] ^$^ x [38] ^$^							**O** [41] ^$^
*Lactobacillus acidophilus* NCFM + *Bifidobacterium lactis* Bi-07	Astro’s *BioBest*	Yogurt	1 × 10^9^	1 × 10^10^	6 months	o [42] ^$^						x [42] ^$^				
*Lactobacillus acidophilus* NCFM	President’s Choice’s *ProAdvantage†*	Yogurt	1 × 10^9^	1 × 10^10^	6 months	o [42] ^$^						x [42] ^$^				

X = beneficial effects observed in healthy adults; x = beneficial effects observed in healthy children, O = studies that have investigated this outcome and have found no significant effect in adults, o = studies that have investigated this outcome and found no significant effect in children, ^$^ = indicates that the research was funded by the company that uses that particular strain in their products. A blank square indicates that no research investigating the effects of that strain/strain combination was identified during the systematic review of all literature published up to 21 July 2016, as described in the methods. All effects reported in this table were found in healthy populations that were not diagnosed with a chronic disease or condition. Definition of health effects: Constipation = improved stool frequency, consistency, or condition; Acute diarrhea = decreased incidence or severity of acute diarrhea; Antibiotic-associated diarrhea = decreased incidence of antibiotic-associated or *Clostridium difficile*-associated diarrhea; Digestive symptoms = decreased abdominal pain/discomfort, bloating, flatulence, or overall GI well-being; Glycemic control = improved fasting glucose, insulin, HbA1c (marker of long-term glycemic control), or HOMA-IR (measure of insulin sensitivity); *Helicobacter pylori* eradication = enhanced eradication of *Helicobacter pylori* infections; Immunity = decreased incidence and/or duration of common infectious diseases, including fever, cough, common respiratory infections (rhinitis, sore throat), common gastrointestinal infections (gastroenteritis, vomiting), asthma, or days missed from school; Infant breastfeeding outcomes = infants (2–6 months old) of mothers who consume this strain while breastfeeding had decreased incidence of gastrointestinal episodes and lower medication-use rates; Inflammation = decreased levels of inflammatory markers (ex. C-reactive protein); Lipids = decreased serum total cholesterol, low density lipoprotein (LDL), triglyceride levels, or increased high density lipoprotein (HDL); Oral health = decreased levels of cavity causing bacteria. * CFU = colony forming units. ** Iogo’s *Probio* reported two strains on its label in 2013 (*Bifidobacterium lactis* BB12 + *Lactobacillus acidophilus* LA-5) and only one strain on its label in 2016. † These products were available in 2013 but may no longer be available in the Canadian market. Note: All cited references were deemed to be of high quality according to Health Canada’s quality appraisal tool for intervention studies [18].

**Table 2:** Results of the review of randomized controlled trials investigating the health effects of probiotic strains found in the Canadian food supply ^1^.

Strain	Study, Country (Year)	Population (*n*)	Probiotic Dosage (CFU per day)	Study Duration	Outcome Measures (Primary and Secondary)	Statistically Significant Effects (Relative to Placebo Group)	Funding Source
*B lactis* BB12 + *L acidophilus* LA-5	Ivey et al. [21] Australia (2014)	Overweight adults *n* = 156	3 × 10^9^	6 weeks	**Primary:** Glycemic control (fasting blood glucose, insulin, HbA1c, and HOMA-IR)	Increased HOMA-IR (worsened insulin sensitivity)	Sir Charles Gairdner Hospital
	Sadrzadeh-Yeganeh et al. [23] Iran (2010)	Females *n* = 90	3.9 × 10^7^	6 weeks	**Primary:** Serum total cholesterol, HDL, LDL, and triglycerides	No observed effects	Tehran University Grant
	Ivey et al. [22] Australia (2015)	Overweight adults *n* = 156	3 × 10^9^	6 weeks	**Primary:** Blood pressure, total cholesterol, HDL, LDL, and triglycerides	No observed effects	Sir Charles Gairdner Hospital
	deVrese et al. [20] Germany (2011)	H pylori infected adults *n* = 88	5 × 10^6^	5 weeks	**Primary:** *Helicobacter pylori* activity; **Secondary:** Frequency, intensity and duration of abdominal pain; stool frequency/consistency; duration of diarrhea episodes; IBS symptoms; orofecal transit time	Decreased duration of antibiotic-associated diarrhea episodes	Chr. Hansen GmbH J. & Co., KG, NOM AG ^$^
	Ashwin et al. [24] India (2015)	Children *n* = 60	2 × 10^6^	7 days	**Primary:** Salivary levels of streptococcus mutans (a cavity causing bacteria)	Reduced salivary *mutans streptococci*	Funded by study author
	Singh et al. [25] India (2011)	Children *n* = 40	5.4 × 10^7^	10 days	**Primary:** Salivary levels of salivary *mutans streptococci* and *lactobacilli* (cavity causing bacteria)	Reduced salivary *mutans streptococci*	Not disclosed
	Ejtahed et al. [43] Iran (2011)	Type II Diabetics *n* = 64	>1 × 10^9^	6 weeks	**Primary:** Fasting blood glucose, HbA1c, insulin and antioxidant molecules (superoxide dismutase, glutathion peroxidase, catalase activity, malondialdehyde concentration, and total antioxidative status)	Decreased fasting blood glucose and HbA1c; increased activity of superoxide dismutase, glutathoine peroxidase, and total antioxidative status	Iran Dairy Industry ^$^
	Mohamadshahi et al. [44] Iran (2014)	Type II Diabetics *n* = 44	>1 × 10^9^	8 weeks	**Primary:** Serum triglycerides, LDL, HDL, triglycerides, LDL:HDL	Decreased LDL:HDL, increased HDL	Nutrition Disease Research Center
	Ejtahed et al.[45] Iran (2012)	Type II Diabetics *n* = 60	6 × 10^8^	6 weeks	**Primary:** total cholesterol, triglycerides, HDL, LDL, total cholesterol:HDL, LDL:HDL	Decreased total cholesterol, LDL, LDL:HDL and total cholesterol:HDL	Grant from Tabriz University
	Nabavi et al. [46] Iran (2014)	Non-alcoholic fatty liver disease patients *n* = 72	>1 × 10^9^	8 weeks	**Primary:** Blood levels of liver enzymes (alanine aminotransferase and aspartate aminotransferase); fasting blood glucose; total cholesterol, triglycerides, LDL, HDL.	Decreased blood levels of liver enzymes, total cholesterol, triglycerides, and LDL	Nutrition Research Center, Tabriz University
	Tonucci et al. [47] Brazil (2015)	Type II Diabetics *n* = 45	2 × 10^9^	6 weeks	**Primary:** Glycemic control (fasting blood glucose, insulin, HOMA-IR, fructosamine, HbA1c); lipid profile (total cholesterol, LDL, VLDL, triglycerides, total cholesterol:HDL); total antioxidant status and cytokine concentrations (Il-6, Il-10, TNF-α, adiponectin, and resistin); fecal short-chain fatty acids	Decreased fructosamine, LDL, and total cholesterol; significant change in HbA1c	Brazilian Agri-Research; Foundation to Support the State of Miras Gerais
*B. lactis* BB12	Caglar et al. [29] Turkey (2008)	Healthy young adults *n* = 24	5 × 10^8^	10 days	**Primary:** Salivary levels of *mutans streptococci* and *lactobacilli* (cavity causing bacteria)	Decreased salivary *mutans streptococi*	Funded by researchers
	Merenstein et al. [48] USA (2010)	Children *n* = 182	1 × 10^10^	90 days	**Primary:** Missed days of school due to illness; **Secondary:** Diarrhea, stool consistency, respiratory infection, missed parental work, doctor visits, illnesses, and overall parental satisfaction	No observed effects	The Gerber Foundation ^$^
	Merenstein et al. [27] USA (2011)	Healthy children *n* = 172	1 × 10^10^	90 days	**Primary:** Missed days of school due to illness; **Secondary:** Diarrhea, stool consistency, respiratory infection, missed parental work, doctor visits, illnesses	No observed effects	USDA
	Kekkonen et al. [28] Finland (2008)	Healthy adults *n* = 62	3.5 × 10^10^	3 weeks	**Primary:** Blood levels of inflammatory markers including C-reactive protein and cytokines (TNF-α, IL-6, IFN-γ, IL-10)	No observed effects	Resaerch Council Finland and Valio ^$^
*L. acidophilus* NCFM + *B. lactis* Bi-07	Leyer et al. [42] China (2009)	Healthy children *n* = 326	1 × 10^10^	6 months	**Primary:** Frequency and duration of fever, cough, rhinorrhea, vomiting, diarrhea, physicians' visits and antibiotic prescriptions; **Secondary:** School absences	Decreased incidence of fever, cough, rhinorrhea, antibiotic use, and days missed from school. Reduced symptom duration.	Danisco ^$^
*B. lactis* DN-173 010	Pinto et al.[41] Brazil (2013)	Healthy adults *n* = 26	not reported	2 weeks	**Primary:** Salivary levels of cavity-associated microorganisms (*mutans streptococci*, *lactobacilli* and total microorganisms) in saliva	No observed effects	Not Disclosed
	Tabbers et al. [38] Netherlands and Poland (2011)	Constipated children *n* = 159	>8 × 10^9^	3 weeks	**Primary:** Stool frequency; **Secondary:** Stool consistency, frequency of faecal incontinence, pain during defecation, abdominal pain, flatulence	Decreased flatulence	Danone ^$^
	Guyonnet et al. [39] Germany (2009)	Healthy adult women *n* = 192	2.5 × 10^10^	4 weeks	**Primary:** Overall GI well-being (intestinal transit, stool frequency and consistency, abdominal pain/discomfort, bloating, flatulence, stomach rumbling); **Secondary:** Frequency of digestive symptoms including abdominal pain/discomfort, bloating, flatulence, stomach rumbling; stool frequency and consistency; health-related quality of life	Improved overall GI well-being; decreased frequency of flatulence, stomach rumbling, improved stool consistency, and health-related quality of life.	Danone ^$^
	Agrawal et al. [40] United Kingdom (2008)	Adult females with IBS *n* = 34	2.5 × 10^10^	4 weeks	**Primary:** Abdominal distension and bloating; **Secondary:** Orocaecal and colonic transit times; incidence and severity of IBS symptoms (abdominal pain/discomfort, bloating, flatulence); overall IBS symptom severity; time and consistency of bowel movements; feelings of incomplete evacuation at time of stool passage	Decreased maximal abdominal distension, orocaecal and colonic transit times, overall IBS symptom severity, and abdominal pain/discomfort.	Danone ^$^
*L. casei* DN 114-001	Guillemard et al. [35] Germany (2010)	Healthy adult shift workers *n* = 1000	>2 × 10^10^	3 months	**Primary:** Cumulative number of common infectious diseases (CID) (e.g., sore throat, sinusitus, nasal discharge, ear ache, influenza, pneumonia, cough, GI infection, diarrhea, nausea vomiting) **Secondary:** Occurrence of having at least one CID: time to first CID, severity, duration, cumulated duration; occurrence and duration of fever, sick days, medication use	Decreased occurrence and time to first CID; decreased duration of fever; decreased cumulative number of CIDs (post-hoc analysis)	Danone ^$^
	Merenstein et al. [26] USA (2010)	Healthy children *n* = 638	>2 × 10^10^	3 months	**Primary:** Change in behaviour due to illness (e.g., missed school, missed sports activity); incidence of common infectious diseases (CIDs) **Secondary:** Absences from daycare or school, missed parental work, days with diarrhea, vomiting, stomach pain, constipation, runny nose, cough, decreasing appetite, fever, rash, medication use	Decreased incidence of CID	Danone ^$^
	Guillemard et al. [36] France (2009)	Elderly adults *n* = 1072	>2 × 10^10^	3 months	**Primary:** Cumulative number of all common infectious diseases (CID) **Secondary:** The occurrence of CID (defined as the number of subjects experiencing at least one CID), duration of CID (cumulative and per episode), time to first CID, severity of CID, fever associated with CID, occurrence or duration of medication use	Decreased duration of CID episodes and cumulative duration of CID	Danone ^$^
	Sykora et al. [34] Czech Republic (2005)	Children w/H Pylori *n* = 86	1 × 10^10^	14 days	**Primary:** Eradication rate of *Helicobacter pylori* infection	Increased *Helicobacter pylori* eradication rates	Ministry of Health and Danone ^$^
	Ortiz-Andrellucchi et al. [37] Spain (2008)	Breastfeeding infants *n* = 104	3 × 10^10^	6 weeks	**Primary:** Immunomodulatory molecules in breast milk (not included in this review) **Secondary:** Infant growth and weight; incidence of gastrointestinal episodes, respiratory symptoms, medication use, allergies and dermatitis	Reduced incidence of gastrointestinal episodes and lower rate of medication use in infants	Danone ^$^
	Agarwal et al. [31] India (2002)	Children *n* = 150	2–3 × 10^10^	9 months	**Primary:** Duration of acute diarrhea	Decreased duration of acute diarrhea	Not Disclosed
	Hickson et al. [33] United Kingdom (2007)	Elderly in-patients *n* = 137	2 × 10^10^	2 weeks	**Primary:** Incidence of antibiotic-associated diarrhea and *Clostridium difficile* associated diarrhea	Decreased incidence of antibiotic- and *Clostridium*-associated diarrhea	Danone ^$^
	Giovannini et al. [30] Italy (2007)	Children with asthma/rhinitis *n* = 187	1 × 10^10^	12 months	**Primary:** Episodes and duration of asthma and rhinitis (runny/stuff nose) **Secondary:** Episodes and duration of abdominal symtoms, diarrhea and fever	Decreased asthma and rhinitis episodes, decreased duration of diarrhea in children with rhinitis	Danone ^$^
	Giralt et al. [49] Spain (2008)	Gynecological cancer patients *n* = 85	2.8 × 10^10^	6 months	**Primary:** Frequency and severity of radiation induced diarrhea **Secondary:** Time to the development of diarrhea, stool consistency	Improved stool consistency	Danone ^$^

^1^ All probiotic strains in the Canadian food supply were recorded and a systematic review of their health effects was conducted. All literature published up to 21 July 2016 was included, as described in the methods. All studies included in the review were deemed to be of a ”high quality” according to Health Canada’s quality appraisal tool for intervention studies and thus are considered eligible to substantiate a health claim [18]. ^$^ Indicates that funding was provided by the food industry HbA1c = hemoglobin A1c, a long-term measure of glycemic control; HOMA-IR = a measure of insulin sensitivity; LDL = low-density lipoprotein; HDL = high-density lipoprotein; VLDL = very low-density lipoprotein; IBS = irritable bowel syndrome; CID = common infectious diseases.
